# Supplementary figures and images for: The feasibility of predicting ground reaction forces during running from a trunk accelerometry driven mass-spring-damper model
Source: PeerJ. 2018 Dec 20;6:e6105. doi: 10.7717/peerj.6105 (PMC6304261; doi:10.7717/peerj.6105)

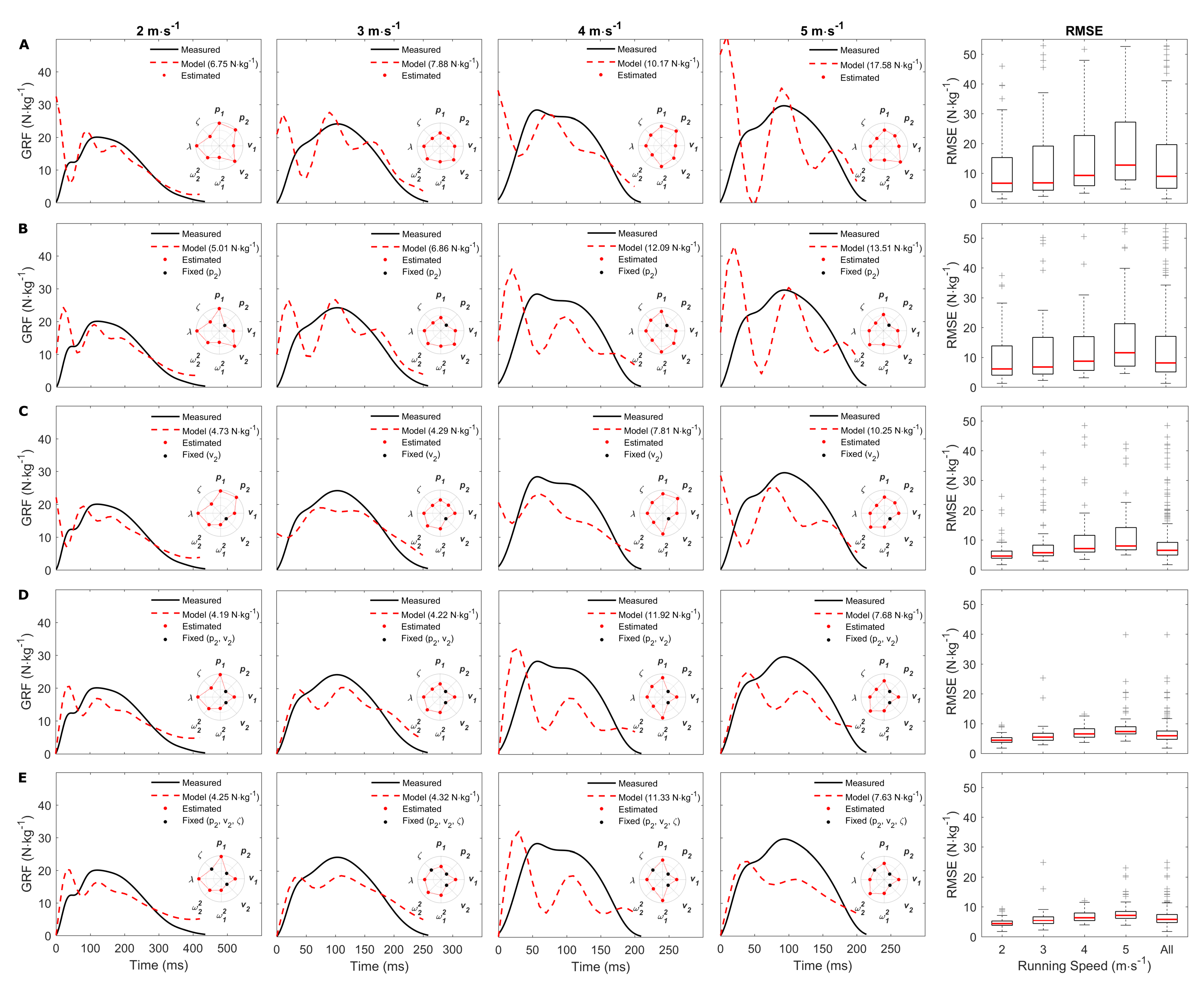

Supplement: Figure S1 — Representative examples of a single stride of the predicted GRF for the four running speeds and boxplots displaying the RMSE median, and 25th and 75th interquartile range when selected model parameters were kept constant. The polar plots display the estimated model parameters (in unscaled values) from the representative examples. When all parameters were estimated from TrunkAcc (A), when p2 was kept constant at 0.00 m for all trials (B), when v2 was kept constant at −0.02 m s −1 (C), when both p2 and v2 were kept constant (D) and finally when p2, v2 and ζ (0.36 au) were kept constant for all trials (E). Extreme outliers were removed from the boxplots. Notice the effect of keeping v2, and both p2 and v2 constant had on the median RMSE and variation in RMSE within and across running speeds, though the predicted GRF waveforms were still unsatisfying. [file peerj-06-6105-s001.png]

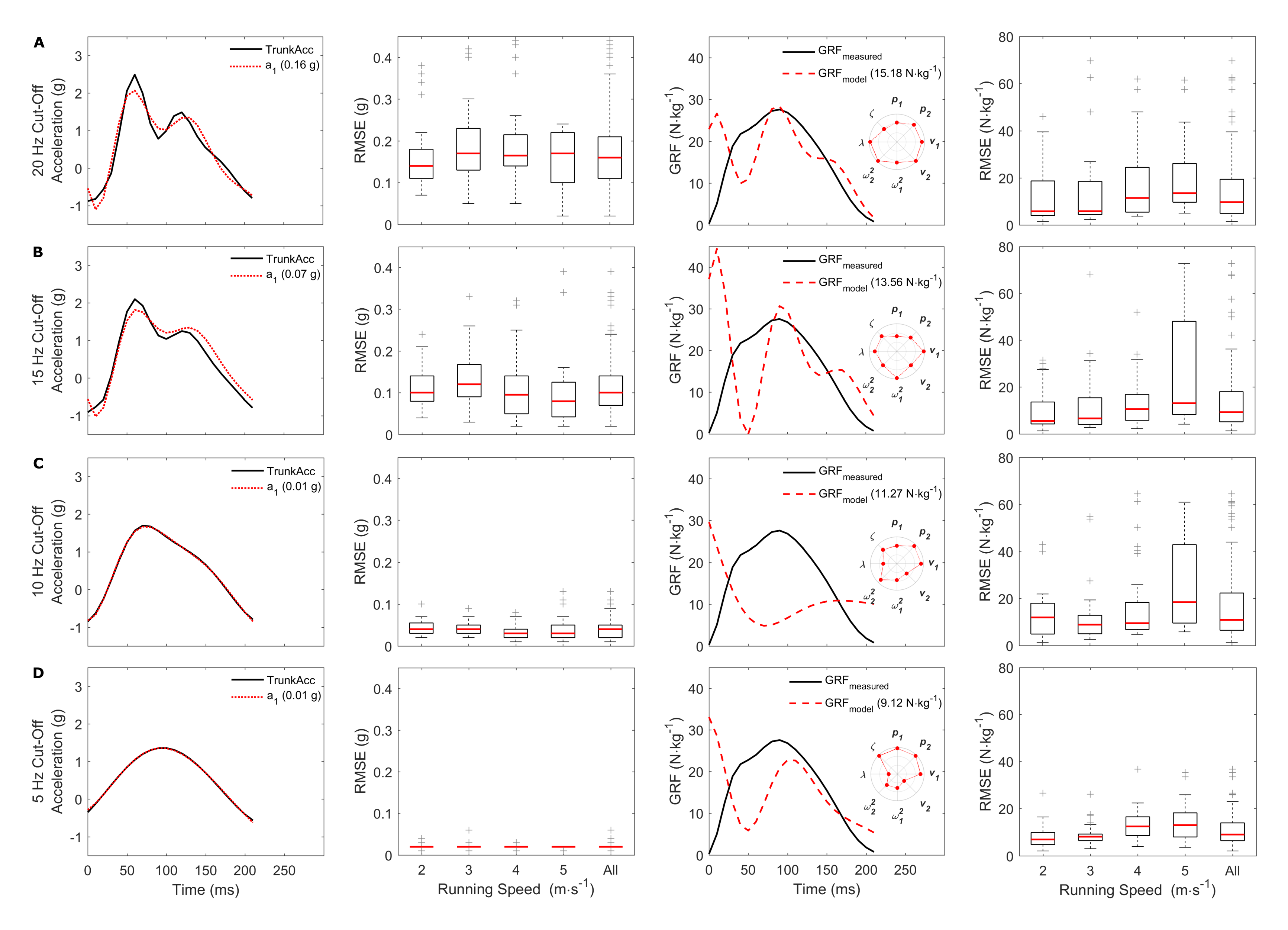

Supplement: Figure S2 — A 4th order recursive Butterworth low-pass filter with a cut-off frequency of 20 Hz (A), 15 Hz (B), 10 Hz (C) or 5 Hz (D) was applied to TrunkAcc from 10 participants (age 21 ± 2 years, height 176 ± 8 cm, mass 76 ± 10 kg) to explore the effect of filter cut-off frequency on model parameter estimation and GRF predictions. Each row display a single stride example of the TrunkAcc fitting; boxplot of the median RMSE, and 25th and 75th interquartile range of the TrunkAcc fitting; a single stride example of the predicted GRF with a polar plot of the estimated model parameters in scaled dimensionless values; and boxplot of the median RMSE, and 25th and 75th interquartile range of the predicted GRF. The example trial displayed is for a running speed of 4 m s−1. Notice how the filter cut-off frequency improved the RMSE of the TrunkAcc fitting, whilst it only had minor influence on the RMSE of the predicted GRF. [file peerj-06-6105-s002.png]
